# Pronounced and prevalent intersexuality does not impede the ‘Demon Shrimp’ invasion

**DOI:** 10.7717/peerj.757

**Published:** 2015-02-10

**Authors:** Amaia Green Etxabe, Stephen Short, Tim Flood, Tim Johns, Alex T. Ford

**Affiliations:** 1 Institute of Marine Sciences, School of Biological Sciences, University of Portsmouth, Portsmouth, United Kingdom; 2 Environment Agency, Howbery Park, Wallingford, Oxfordshire, UK

**Keywords:** Amphipoda, Crustacea, Invasive species, Intersexuality, Microsporidia

## Abstract

Crustacean intersexuality is widespread and often linked to infection by sex-distorting parasites. However, unlike vertebrate intersexuality, its association with sexual dysfunction is unclear and remains a matter of debate. The ‘Demon Shrimp,’ *Dikerogammarus haemobaphes*, an amphipod that has invaded continental waterways, has recently become widespread in Britain. Intersexuality has been noted in *D. haemobaphes* but not investigated further. We hypothesise that a successful invasive population should not display a high prevalence of intersexuality if this condition represents a truly dysfunctional phenotype. In addition, experiments have indicated that particular parasite burdens in amphipods may facilitate invasions. The rapid and ongoing invasion of British waterways represents an opportunity to determine whether these hypotheses are consistent with field observations. This study investigates the parasites and sexual phenotypes of *D. haemobaphes* in British waterways, characterising parasite burdens using molecular screening, and makes comparisons with the threatened *Gammarus pulex* natives. We reveal that invasive and native populations have distinct parasitic profiles, suggesting the loss of *G. pulex* may have parasite-mediated eco-system impacts. Furthermore, the parasite burdens are consistent with those previously proposed to facilitate biological invasions. Our study also indicates that while no intersexuality occurs in the native *G. pulex*, approximately 50% of *D. haemobaphes* males present pronounced intersexuality associated with infection by the microsporidian *Dictyocoela berillonum*. This unambiguously successful invasive population presents, to our knowledge, the highest reported prevalence of male intersexuality. This is the clearest evidence to date that such intersexuality does not represent a form of debilitating sexual dysfunction that negatively impacts amphipod populations.

## Introduction

**Figure 1:**
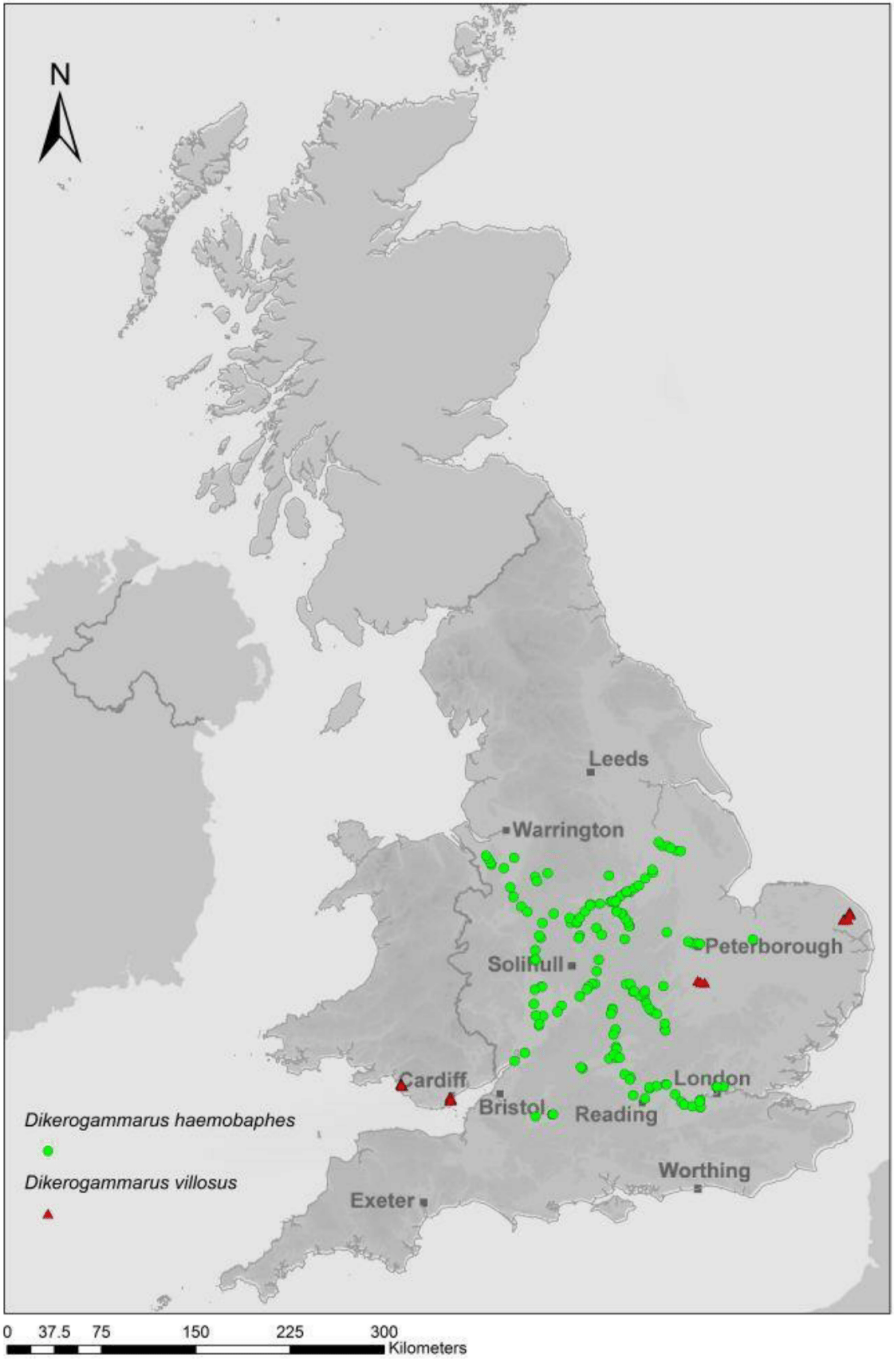
Recent confirmed reports of *D. haemobaphes* (green triangles) and *D. villosus* (red circles) in UK waterways (EA–unpublished data January 2014; image courtesy of SE Environment Agency).

*Dikerogammarus haemobaphes* (Eichwald, 1841), an effective predatory amphipod from the Ponto-Caspian (Bacela-Spychalska & Van Der Velde, 2013), has spread through Europe and is now recognised as an extremely successful invader of British waterways (Green Etxabe & Ford, 2014). *D. haemobaphes*, also known as the ‘demon shrimp,’ invaded the British Isles more recently than the infamous ‘killer shrimp’ (*Dikerogammarus villosus*, Sowinsky, 1894) (Macneil et al., 2010) but is already more widespread (Fig. 1). Amphipods harbour many parasites that can drastically impact host populations by influencing the health, behaviour, reproduction and sex determination of their host (Hatcher & Dunn, 2011; Bacela-Spychalska et al., 2012). The invasive *D. haemobaphes*, therefore, could not only outcompete and prey on native amphipod species, but also introduce parasites into their new habitats. Screening parasites in invasive and native amphipod species associated with a rapid and on-going invasion will test hypotheses that particular parasitic burdens impact invasion success MacNeil et al., 2003a; Hatcher & Dunn, 2011; Hatcher, Dick & Dunn, 2014.

Some amphipod-infecting parasites maximise their transmission via the host’s progeny by converting males into reproductive females (Ford, 2012). Infection by such parasites results in sex-biased populations (Terry et al., 2004) and, in cases of incomplete conversion, intersexuality, where individuals present secondary sex characteristics of both genders (Ford, 2012). Intersex phenotypes are found in a range of animals (Matthiessen & Gibbs, 1998; Harris et al., 2011; Hayes et al., 2002), including crustaceans (Ginsburger-Vogel, 1991; Bishop, 1974; Ford, 2012), where they are linked to parasitic infection (Li, 2002; Short et al., 2012a) and environmental conditions (Dunn, Mccabez & Adams, 1996), as well the direct (Short et al., 2012b) and indirect (Jacobson et al., 2010) influence of contaminant exposure. In cases of parasitic infection, an incomplete conversion is thought to occur due to insufficient parasite burden, suboptimal conditions, or effective host responses (Dunn & Rigaud, 1998; Kelly, Dunn & Hatcher, 2002; Short et al., 2014). Current evidence suggests that the impact of female intersexuality is subtle (Ford et al., 2003; Kelly, Hatcher & Dunn, 2004) or effectively non-existent (Glazier, Brown & Ford, 2012), and that the female intersexuality observed in *D. haemobaphes* successfully invading Polish waterways (Bacela, Konopacka & Grabowski, 2009) is consistent with these hypotheses.

Male intersexuality is widespread in amphipods; however, our understanding of its reproductive consequences is poorly understood relative to vertebrates (Harris et al., 2011). The extents of morphological and behavioural changes (McCurdy et al., 2008; Yang, Kille & Ford, 2008) have led to the suggestion (Yang, Kille & Ford, 2008; Ford, 2012) that the impact of crustacean male intersexuality may be similar to that seen in vertebrates (Harris et al., 2011). Despite some evidence of intersexuality in invasive *D. haemobaphes* (Bacela, Konopacka & Grabowski, 2009), sexual phenotypes in this species have not been studied, even though notable levels of intersexuality in the unambiguously successful invading population would reveal considerable insight into the consequences of intersexuality for wild crustacean populations.

This study investigates the sexual phenotypes and parasites of *D. haemobaphes* and the native *Gammarus pulex* (Linnaeus, 1758) at multiple locations in British waterways to give insights into this rapidly invading species and expand our understanding of crustacean intersexuality.

## Methods

### Specimen characterisation

Amphipods were collected from Wallingford Bridge and Bell Weir, U.K. Amphipods were categorised into species and phenotypes: males, females, intersex males and intersex females. Intersex males were identified by genital papillae, between pereonite 7 and pleonite 1, in conjunction with rudimentary oostegites. Intersex females were identified by oostegites in conjunction with secondary genital papilla/e. Animals from each phenotype were measured from antennal joint to telson to obtain body length (ImageJ, v1.4u4) and comparisons were made using analysis of variance (ANOVA) with the post hoc Tamhanes-T2 test (SPSS v21).

### Scanning electron microscopy

Specimens of *D. haemobaphes* were taken through transitional steps (100% ethanol to 100% hexamethyldisilazane, HMDS) then evaporated to dryness. The dry samples were mounted on SEM stubs, sputter-coated with gold-palladium and examined using a scanning electron microscope (JSM-606LV; JEOL, Welwyn Garden City, Herts, UK) operating in high vacuum mode with a secondary electron detector active at an acceleration voltage of 10 kV. Images were cropped and colourised using Adobe Photoshop (CS5v12).

### PCR screen

DNA was purified from internal animal tissue (excluding gut) or eggs using the DNeasy Blood and Tissue Kit (Qiagen, North Manchester, UK). Samples were screened using previously described PCR primers for general parasites (Table 1). PCR reactions were performed in 25 µl volumes containing 10 ng of DNA as template, 1 U of Taq polymerase (Promega, Southampton, Hampshire, UK), 5 µl of 5× PCR buffer, 1.25 mM MgCl_2_ and 0.4 mM of each corresponding primer. Quality of the DNA samples were analysed using the primers 1073F and 18SR (Table 1) which amplified a 867bp product of the host 18S ribosomal RNA gene.

**Table 1:** Primers used to conduct parasite screen.

Target	Primer	Sequence	Reference
Microsporidea 16S	V1f	5′-CACCAGGTTGATTCTGCCTGAC-3′	Weiss et al., 1994
	1342AC	5′-ACGGGCGGTGTGTACAAGGTACAG-3′	Yang et al., 2011
Acanthocephala 18S	537F	5′-GCCGCGGTAATTCCAGCTC-3′	Near, Garey & Nadler, 1998
	1133R	5′-CTGGTGTGCCCCTCCGTC-3′	
	1073F	5′-CGGGGGGAGTATGGTTGC-3′	
	18SR	5′-TGATCCTTCTGCAGGTTCACCTAC-3′	
	18SF	5′-AGATTAAGCCATGCATGCGTAAG-3′	
	549R	5′-GAATTACCGCGGCTGCTGG-3′	
Nematode/acanthocephala/apicomplexa	Nem18SlongF	5′-CAGGGCAAGTCTGGTGCCAGCAGC-3′	Wood et al., 2013
	Nem18SlongR	5′-GACTTTCGTTCTTGATTAATGAA-3′	
Paramyxea	Para18SF3	5′-CTACGGCGATGGCAGGTC-3′	Short et al., 2012b
	Para18SR3	5′-GGGCGGTGTGTACAAAGG-3′	
*Wolbachia*	WSPEC-F	5′-CATACCTATTCGAAGGGATAG-3′	Werren & Windsor, 2000
	WSPEC-R	5′-AGCTTCGAGTGAAACCAATTC-3′	

### Sequence identification

PCR products were analysed using agarose gel electrophoresis containing 1x GelGreen™ (Cambridge Bioscience, UK) for the presence of bands potentially representing amplified parasite sequences. Individual bands were isolated and DNA extracted using the QIAquick Gel Extraction Kit (Qiagen, North Manchester, UK) and sequenced (Source Bioscience, Cambridge, UK), before a BLAST analysis was performed against sequences stored in GenBank (NCBI).

## Results

### Sexual phenotypes

**Figure 2:**
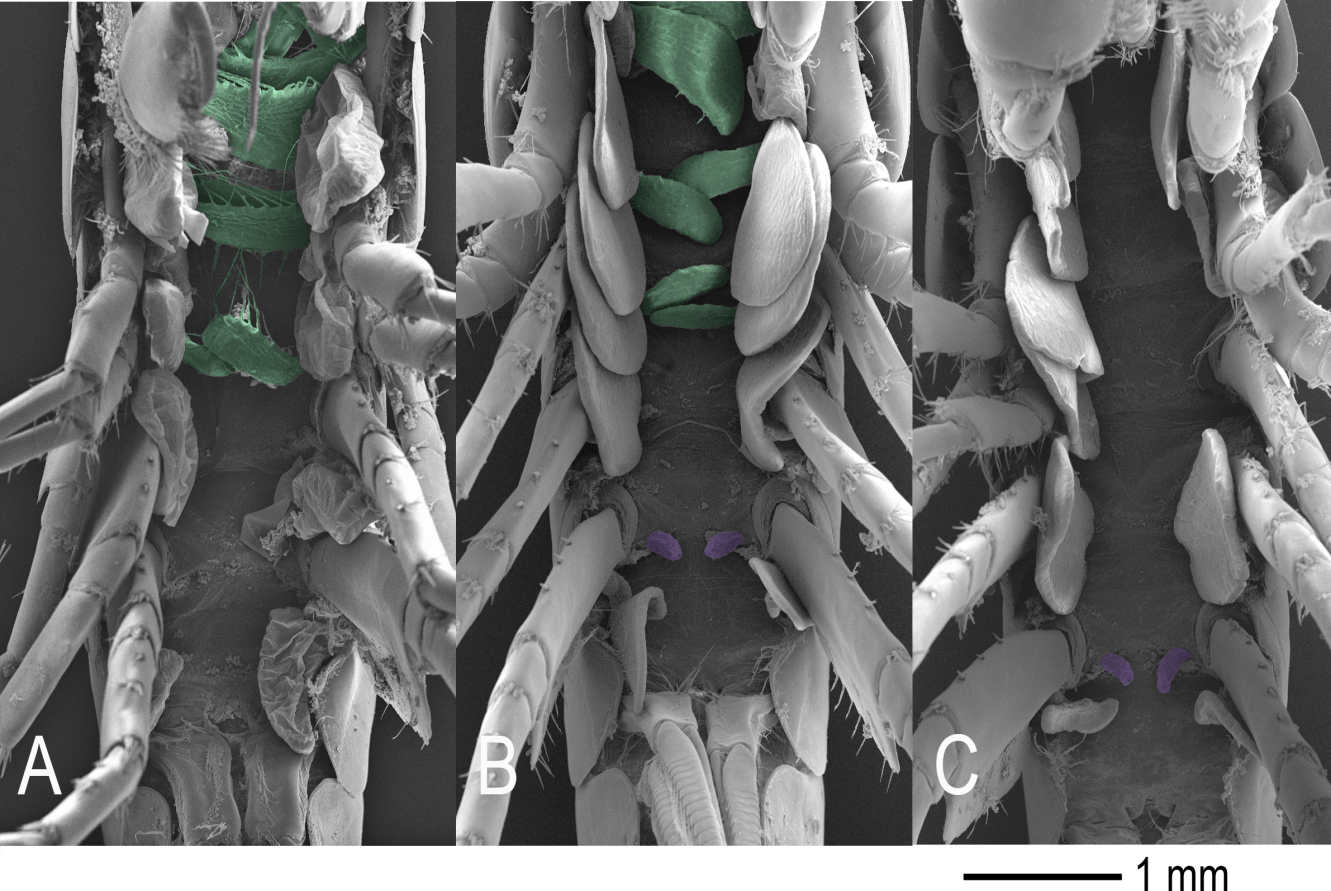
External sexual phenotypes. (A) Normal female *D. haemobaphes* with only oostegites (green). (B) Intersex male *D. haemobaphes* specimen presenting genital papillae (purple) alongside oostegites (green) with rudimentary setae. (C) Normal male *D. haemobaphes* with only genital papillae (purple).

**Figure 3:**
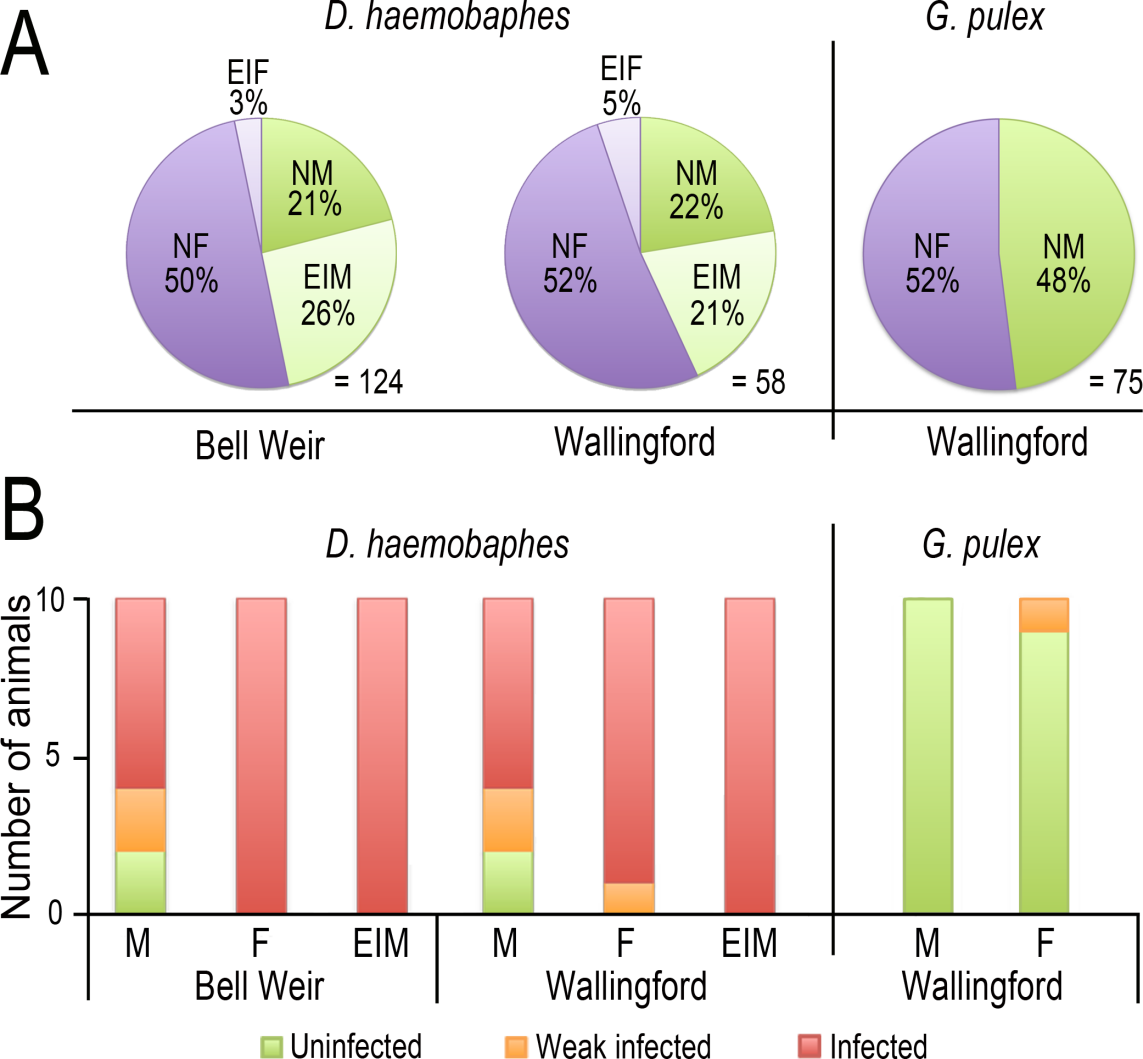
Frequency of sexual phenotypes and prevalence of *D. berillonum* infection. (A) Sexual phenotypes found in two *D. haemobaphes* populations and *G. pulex*. (B) Infection of *D. berillonum* found in *D. haemobaphes* and *G. pulex* found in both sites (NF, Normal Female; EIF, External Intersex Female; NM, Normal Male; EIM, External Intersex Male).

Pronounced male intersex phenotypes were found in *D. haemobaphes* at both sites, with most specimens displaying well-developed oostegites with visible setae (Fig. 2). Almost half the male population presented intersex characteristics at both locations and very few cases of female intersexuality were observed (Fig. 3A). *G. pulex* was only found in conjunction with *D. haemobaphes* at one sampling site and no intersex phenotypes were found (Fig. 3A). Significant differences were found in lengths of *D. haemobaphes* phenotypes (*F* = 3.885, df = 2, *p* = 0.023) where normal males (14.85 mm ± 3.65, *N* = 32) are significantly larger (*p* = 0.04) than females (13.01 mm ± 2.29, *N* = 52). However, there is no significant difference between intersex males (13.57 mm ± 3.10, *N* = 37) and either females (*p* = 0.735) or males (*p* = 0.328), therefore forming an intermediate size.

### Parasite screening

Screening of *D. haemobaphes* and *G. pulex* populations revealed evidence of infection by several parasites (Table 2). All *D. haemobaphes* females and intersex males were found infected with *D. berillonum*, with one female weakly infected (Fig. 3B), as previous defined (Yang et al., 2011). The majority of males were also infected, although more weak infections were found (Fig. 3B). This pattern of *D. berillonum* infection was consistent at both collection sites and when combined in a Fishers Exact test (two-tailed) reveal a significant difference in the level of infection between normal and intersex males (*p* = 0.003 using strong infections only, *p* = 0.02, using weak and strong infections). To confirm vertical transmission, the broods of ten infected females were also tested and all were infected by *D. berillonum*. Only one case of weak *D. berillonum* infection was found in *G. pulex* (Fig. 3B).

**Table 2:** A screen of parasites using a subsample of the *D. haemobaphes* and *G. pulex* populations revealed infection by a variety of parasites. Strong infection as defined by previous studies (Yang et al., 2011).

Amphipod	Phylum of isolated parasite	Number of strongly infected animals	Length of ribosomal sequence	Primers used for amplification	GenBank accession of isolated sequence	Closest identity using a BLAST	GenBank accession of closest match	% Identity
***D. haemobaphes***	Nematoda	11/60	472bp	537F & 1133R	KM486061	*Hysterothylacium deardorffoverstreetorum*	JF718550	100%
	Microsporidia	51/60	1148bp	V1f & 1342AC	KM486059	*Dictyocoela berillonum*	KF830272	99.9%
***G. pulex***	Acanthocephala	3/20	547bp	537F & 1133R	KM486063	*Echinorhynchus gadi* *Echinorhynchus truttae*	AY830156	98%
	Microsporidia	10/20	1135bp	V1f & 1342AC	KM486060	*Pleistrophora hippoglossoideos* *Pleistrophora typicalis* *Pleistrophora mulleri*	EF119339	99.6%
	Apicomplexa	10/20	402bp	537F & 1133R	KM486064	*Mattesia geminate*	AY334568	90.2%

## Discussion

Our screen of invasive and native species associated with an extremely successful, and ongoing, amphipod invasion reveals parasitic-profiles strikingly consistent with hypotheses that particular parasitic burdens influence the dynamics of biological invasion (MacNeil et al., 2003a; Hatcher & Dunn, 2011; Hatcher, Dick & Dunn, 2014). The native *G. pulex* are infected with a microsporidian of the genus *Pleistophora*, which include behaviour-altering species known to increase the likelihood of predation on native amphipods and reduce their predatory behaviour when interacting with invaders (MacNeil et al., 2003a; Fielding et al., 2005). Sequences were also found for an acanthocephalan, most likely *Echinorhynchus truttae*. This species can both reduce its host’s predatory behaviour and increase vulnerability to predation by fish (Fielding et al., 2003; MacNeil et al., 2003b; Lagrue, Güvenatam & Bollache, 2013). Consequently, the parasite burden of *G. pulex* may facilitate invasion of *D. haemobaphes* through British waterways by impairing the competitive abilities of the native population, a scenario consistent with recent experiments and population modelling (MacNeil et al., 2003a; Haddaway et al., 2012; Hatcher, Dick & Dunn, 2014). In contrast, the invasive *D. haemobaphes* was almost ubiquitously infected by the vertically transmitted microsporidian *Dictyocoela berillonum*. It is possible the initial invasive population consisted of a small number of infected individuals and the current infection prevalence represents a parasitic founder-effect. Alternatively, given that parasite infection is predicted to influence invasion success (MacNeil et al., 2003a; Fielding et al., 2005; Hatcher, Dick & Dunn, 2014) via trait-mediated effects, it is possible the high prevalence of *D. berillonum* occurs due to a subsequent enhancement in invasive capabilities.

The distinct parasitic profiles of *G. pulex* and *D. haemobaphes* may have ecological impacts. Our results suggest the eradication of native *G. pulex* would lead to the removal of a pleistophoran microsporidian from the ecosystem potentially capable of causing disease in fish (Lom & Nilsen, 2003) and an acanthocephalan indistinguishable from *E. truttae* (García-Varela & Nadler, 2005). Although *E. truttae* infection in fish does not appear to cause morbidity (Dorucu et al., 1995), infected amphipods are more vulnerable to fish predation due to altered habitat usage (MacNeil et al., 2003b; Lagrue, Güvenatam & Bollache, 2013). Therefore, the loss of this parasite may alter prey abundance, even if the overall amphipod biomass is maintained following the displacement of *G. pulex*.

The sexual phenotype survey revealed that while no intersexuality was evident in *G. pulex*, the invasive *D. haemobaphes* presents striking levels of pronounced male intersexuality, where males exhibit unambiguous oostegites possessing rudimentary seta, and their size is not significantly different from males or females. In contrast, the low levels of female intersexuality in *D. haemobaphes* were much like those previously reported in Polish waters (Bacela, Konopacka & Grabowski, 2009). To our knowledge, this is the highest prevalence of male intersexuality recorded in an amphipod population (McCurdy et al., 2004; Ford & Fernandes, 2005; Short et al., 2012b; Yang et al., 2011) and is the first evidence clearly linking *D. berillonum* with amphipod intersexuality (Terry et al., 2004; Yang et al., 2011). Other *Dictyocoela* species have been linked to both abnormal sexual phenotypes and female-biased sex ratios (Terry et al., 2004; Short et al., 2012a), however, the lack of female-bias in *D. haemobaphes* suggests *D. berillonum* is unable to fully convert males in to females. This could result from sub-optimal environmental conditions impacting the efficacy of conversion or the consequence of *D. berillonum* infecting an unfamiliar host. Whatever the cause, the *D. haemobaphes* intersexuality is of interest. The association between male intersexuality and sexual dysfunction is, despite recent molecular advances (Short et al., 2014), still poorly understood. The functional impact of *D. haemobaphes* intersexuality is unclear but must incur some form of cost, even if the production of non-functional oostegites on intersexes is merely reducing the resources available for normal growth and reproduction. It is also possible that intersexuality is the outward manifestation of more serious sexual dysfunction. Lower sperm counts have been reported in intersex males of *Echinogammarus marinus* (Yang, Kille & Ford, 2008) and in *Corophium volutator*, also females mating with intersex males produce smaller broods (McCurdy et al., 2004). Furthermore, intersexuality may be associated with behavioural changes. Gammarid amphipods mate after a period of mate-guarding and a reduced capacity of intersex males to initiate or maintain this behaviour could also impact reproductive success. The plausibility of such altered behaviours is made more likely given numerous behavioural changes observed in *C. volutator* intersexes (McCurdy et al., 2008). Investigation of *D. haemobaphes* reproductive function and behaviour will help determine the extent of dysfunction associated with the intersexuality.

Although the observed intersexuality will incur some cost, the fact that such high levels of pronounced intersexuality has not impeded a successful amphipod invasion is the strongest evidence to date that crustacean male intersexuality is not, in any meaningful sense, equivalent to vertebrate male intersexuality, which is commonly associated with serious sexual dysfunction (Jobling et al., 1998; Harris et al., 2011; Kidd et al., 2007). Furthermore, our findings are consistent with experimentally generated hypotheses that certain parasitic burdens facilitate biological invasions.

## Additional Information and Declarations

### DNA Deposition

The following information was supplied regarding the deposition of DNA sequences:
KM486061

KM486059

KM486063

KM486060
KM486064.
